# Increased Yield and High Resilience of Microbiota Representatives With Organic Soil Amendments in Smallholder Farms of Uganda

**DOI:** 10.3389/fpls.2021.815377

**Published:** 2022-02-02

**Authors:** Martina Köberl, Peter Kusstatscher, Wisnu Adi Wicaksono, Samuel Mpiira, Francis Kalyango, Charles Staver, Gabriele Berg

**Affiliations:** ^1^Institute of Environmental Biotechnology, Graz University of Technology, Graz, Austria; ^2^National Agricultural Research Laboratories, National Agricultural Research Organisation, Kampala, Uganda; ^3^Bioversity International, Montpellier, France

**Keywords:** *Gammaproteobacteria*, banana fruit microbiome, organic soil amendments, manure, mulch, smallholder farms, plant-associated microbiota

## Abstract

Organic matter inputs positively affect soil fertility and quality but management effects on the soil and plant microbiome are less understood. Therefore, we studied the response of microbial colonization of the East African highland banana cultivar “Mpologoma” (AAA genome) under different mulch and manure treatments on three representative smallholder farms in Uganda. In general, the gammaproteobacterial community appeared stable with no significant response to organic matter inputs after 24 months of treatment. Significant differences (*p* < 0.05) in the plant-associated carpo-, phyllo-, and rhizosphere microbial community composition and diversity were found among individual sampled farms, independent of added soil inputs. Across farms, banana fruit harbored a richer and more balanced gammaproteobacterial community than the rhizo- and endospheres. Gammaproteobacterial beta diversity was shaped by the microenvironment (44%) as well as the sampling site (4%). Global effects of treatments in the rhizosphere analyzed using linear discriminant analysis effect size showed significantly enriched genera, such as *Enterobacter*, under manure and mulch treatments. As shown in previous works, bunch size and total yield were highly increased with manure and mulch, however, our results highlight general short-term microbial stability of Ugandan banana cropping systems with increases in the gammaproteobacterial community.

## Introduction

The plant and its highly diverse plant-associated microbial community, including bacteria, archaea, fungi, and protists, are nowadays recognized as holobiont (Vandenkoornhuyse et al., 2015). Essential functions are outsourced to symbiotic microbiota partners, and therefore plant-associated microbiomes play a key role in plant survival and health (Gilbert et al., 2012; Guttman et al., 2014). The species- and habitat-specific plant microbiota contribute to multiple aspects in functioning of the plant holobiont, such as (i) seed germination and growth, (ii) nutrient supply, (iii) resistance against biotic and abiotic stress factors, and (iv) production of bioactive metabolites (Berg et al., 2016, 2020). The plant microbiome and its interaction with the host were identified as an important research focus for sustainable crop production and has been identified as key to the next, and sustainable, agricultural revolution (Schlaeppi and Bulgarelli, 2015; Bender et al., 2016). Despite ever-increasing interest in microbial strains as bio-pesticides, they are, however, far from overtaking chemical pesticides used in agriculture (Weller, 2007; Berg et al., 2013). In addition to microbial inoculants, an extensive list of microbiome management strategies and products were developed in agriculture including (i) microbiome transplants (straw dung, manure, mulch, and biodynamic additives), (ii) microbial and plant extracts as well as (iii) methods to change environmental conditions (Berg et al., 2020, 2021; French et al., 2021). Nevertheless, many parts of the underlying mechanisms are yet not fully understood.

African farming systems largely comprise a tapestry of crops and livestock dominated by smallholders with low crop yields but high biodiversity. All traditional farming systems in East Africa produce a variety of bananas. Bananas are also fruits of global importance with Latin America and the Caribbean (LAC) as the main producers worldwide (Lescot, 2011; FAOSTAT, 2018). Threatened by major *Fusarium wilt* disease outbreaks in the past and a new even more devastating strain currently menacing new areas, the banana industry is always on the look out for ways to control pathogens efficiently with promising advances with microbial-based suppressive soils (Butler, 2013; Dita et al., 2018; Bubici et al., 2019; García-Bastidas et al., 2020). While less is known about the banana fruit microbiome, a study on the rhizosphere and endosphere revealed *Gammaproteobacteria* as an important and dominant group in banana farms in Uganda contributing a third of the total bacterial population (Rossmann et al., 2012). Agroforestry was found to have especially an effect on plant-associated *Gammaproteobacteria* (Köberl et al., 2015). Moreover, *Gammaproteobacteria* are generally an important and dominant group within the plant microbiome comprising a variety of PGPR and biological control agents (e.g., *Pantoea sp., Pseudomonas sp., Serratia sp*.) but also potential plant pathogenic members (e.g., *Xanthomonas sp., Xylella sp., Pseudomonas sp*.) (Eastgate, 2000; Buchholz et al., 2011; Fürnkranz et al., 2012; Rastogi et al., 2012). Hence, this class was identified as a potential health indicator of banana plants (Köberl et al., 2017). Some members are also considered to be opportunistic human pathogens and have been implicated in foodborne diseases and outbreaks (Bloch et al., 2012; Berg et al., 2015); which indicates them, together with their dominant abundance in banana, as a model group of interest.

In this study, the microbial colonization of the East African highland banana cultivar “Mpologoma” (AAA genome) was investigated on three smallholder farms in Uganda from a larger study of plots on 54 farms, in order to reveal differences according to applied organic soil amendments. Different combinations of mulch and manure (mulch, manure, mulch + manure, non-treated control) were investigated. Previous results from the 54 farms have already shown, that shrubs and trees grown on the farms (e.g., *Calliandra, Magnifera, Sesbania*, or *Tithonia*) provide a rich source of nutrients such as nitrogen, potassium, and phosphorus, changing the physiochemical properties (Mpiira et al., 2013; Kongkijthavorn, 2017) and yield (50–100%) (Staver et al., 2015). Moreover, organic matter input to the soil affects biotic factors related to the soil microbiota, such as microbial biomass, microbial diversity, and community structure (Saison et al., 2006; Wallis et al., 2010; Bonilla et al., 2012, 2015). Here, we investigated if (I) specific soil amendments induce significant shifts in the banana gammaproteobacterial community and if effects can be found within (II) specific compartments (carpo-, phyllo-, and rhizosphere) or locations (III). Moreover, we investigate the currently unknown (IV) banana fruit microbiome and whether it reflects soil management practices. This study increases our knowledge of the impact of soil amendments on the plant-associated microbial community. Further studies based on more holistic population microbiology and over longer time periods are, however, necessary to potentially extend the gammaproteobacterial shifts/resilience to the entire microbial community.

## Materials and Methods

### Experimental Design and Sampling Procedure

**Figure 1:**
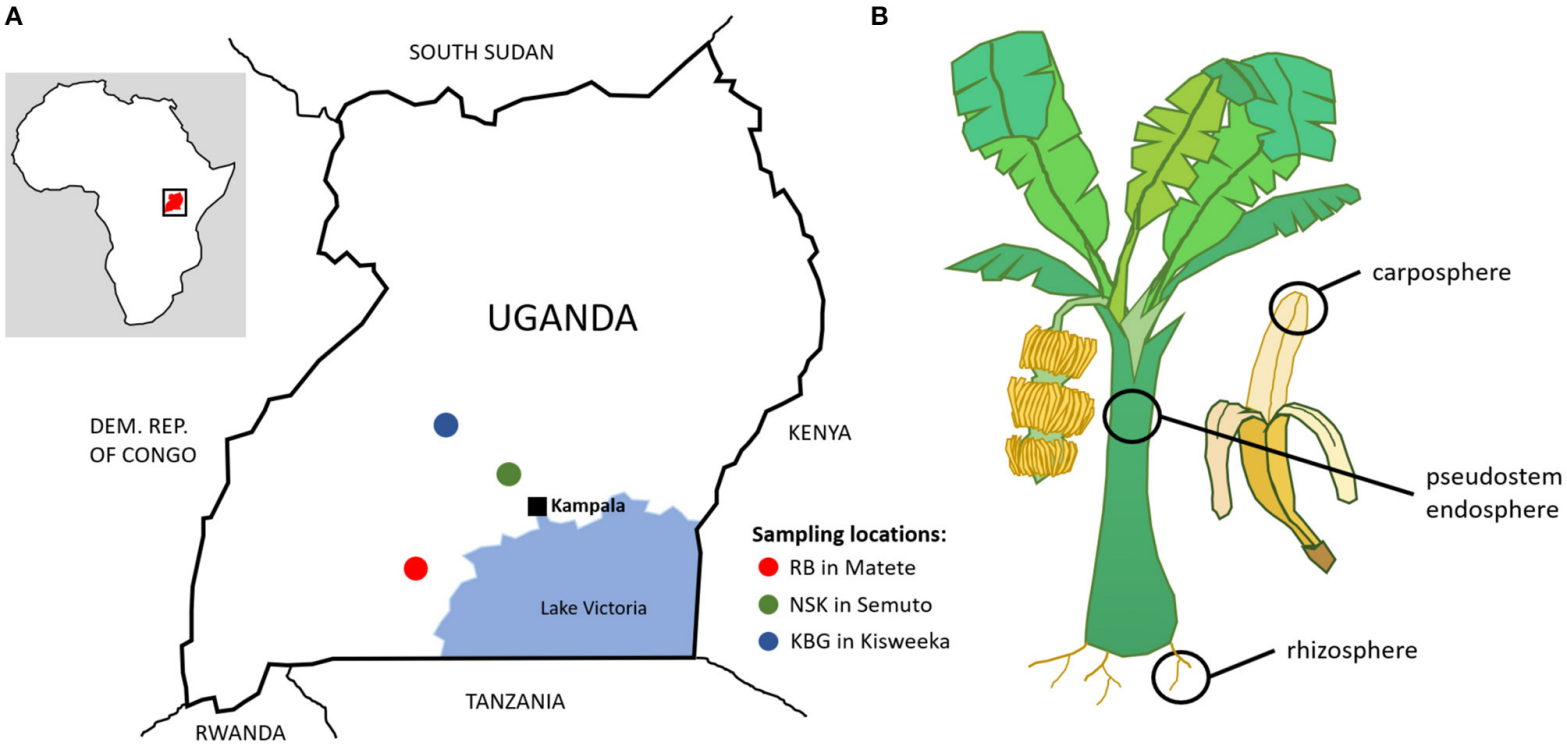
Geographic location of sampling spots in Uganda **(A)** and schematic representation of samples **(B)**.

The rhizosphere, pseudostem endosphere, and carposphere samples were collected from the East African highland banana cultivar “Mpologoma” (AAA genotype) in July 2013. The same set of samples was collected at three smallholder farms in Uganda: RB farm in Matete in the district Sembabule, NSK farm in Semuto in the district Nakaseke, and KBG farm in Kisweeka in the district Kiboga (Figure 1). Soils in the areas are classified as Ferric Acrisols (FAO, 1977) comprising approximately 30% clay (FAO, 2004). Soil pH was 6.1 (NSK), 6.4 (KBG), and 6.6 (RB). Additionally, chemical soil properties were analyzed by (Kongkijthavorn, 2017). The whole region is characterized by a bimodal rainfall pattern, with the first peak from March to June and a second peak from August to December. The annual mean temperature is similar for all the sites around 23°C with 15°C minimum to near 30°C maximum. Plots did not have previous banana cultivation. Four treatments were planted on each farm with 10 plants/treatment—a control and three combinations of mulch and manure (mulch, manure, mulch + manure). In the manure and mulch + manure plots, 10 kg of air-dried cow manure was applied in each banana mat at the time of planting. Farmers followed up with yearly applications of 8–10 kg/plant of goat manure produced on the farm. Farmers used their own sources of mulch to maintain the soil surface covered—maize stover, cut grass, shrubs and tree trimmings, and unconsumed fodder materials from goat feeding. Sampling for microbial communities was completed 2 years after plot establishment. All the plants sampled were at the same physiological stage with a bunch approaching harvest. For each microenvironment and treatment, two replicate composite samples consisting of sub-samples from five plants were taken. Additional information about the region, the banana cropping system, and the use of trees and shrubs to improve banana productivity are given by (Mpiira et al., 2013; Kongkijthavorn, 2017). Banana yield data were previously reported by (Staver et al., 2015).

### Total Community DNA Isolation

For the extraction of metagenomic DNA from the rhizosphere, 5 g of roots with adhering soil were added to 40 ml of sterile 0.85% NaCl solution and mixed for 5 min. Pseudostem samples (5 g) were washed with sterile distilled water, transferred to Whirl-Pak bags (Nasco, Fort Atkinson, Wisconsin, USA), and after 10 ml of 0.85% NaCl were added, homogenized with mortar and pestle. From the banana fruits, 5 g of pulp was washed with sterile distilled water, before homogenization with 10 ml of 0.85% NaCl. From the liquid parts, 4 ml were centrifuged (10 min, 16,000 × *g*, 4°C) and the resulting pellets were stored at −70°C until further processing. DNA was extracted using the FastDNA SPIN Kit for Soil (MP Biomedicals, Solon, Ohio, USA) and quantified using a NanoDrop 2000c spectrophotometer (Thermo Scientific, Waltham, Massachusetts, USA). Metagenomic DNA samples were encoded using abbreviations indicating: (1) microenvironment [R = rhizosphere, PS = pseudostem, C = carposphere (fruit)], (2) farm (RB, NSK, KBG) and (3) organic matter input (mulch, mulch + manure, manure, control = non-treated).

### Gammaproteobacterial 16S rRNA Gene Profiling by Illumina MiSeq Sequencing

For a deep-sequencing analysis of the banana-associated *Gammaproteobacteria* community, the hypervariable V4 region of the 16S rRNA gene was amplified in a nested PCR approach with first a *Gammaproteobacteria*-specific primer pair Gamma395f/Gamma871r (Mühling et al., 2008) and then the universal primer pair 515F/806R (Caporaso et al., 2011), which carried sample-specific barcodes for demultiplexing. The reaction mixture for the first PCR (20 μl) contained 1× Taq&Go (MP Biomedicals, Eschwege, Germany), 2 mM MgCl_2_, 0.1 μM of each primer, and 1 μl (~10 ng) of template DNA dilution (96°C, 4 min; 30 cycles of 96°C, 1 min; 54°C, 1 min; 74°C, 1 min; and elongation at 74°C, 10 min). The second PCR (30 μl) was performed by using 1× Taq&Go, 0.2 μM of each primer, and 1.2 μl from dilutions (1:10^3^) of the first PCR mixtures (94°C, 3 min; 32 cycles of 94°C, 45 s; 60°C, 1 min; 72°C, 18 s; and elongation at 72°C, 10 min). PCR products of three technical replicates per sample were purified by employing the Wizard SV Gel and PCR Clean-Up System (Promega, Madison, WI, USA). Samples were pooled in equimolar concentrations and amplicon libraries were sequenced with a paired-end approach (2 × 300 bp, chemistry v3) using an Illumina MiSeq platform (Eurofins Genomics, Ebersberg, Germany).

### Data Processing

Data analysis was performed by employing QIIME version 1.9.1 (Caporaso et al., 2010b). Joined paired-end reads with more than three consecutive low-quality base calls (Phred quality score ≤ 25) were truncated at the position where their quality began to drop, and only reads with > 75% consecutive high-quality base calls, without any ambiguous characters, and longer than 200 nucleotides in length were retained for further analyses. Demultiplexed high-quality reads were *de novo* clustered into operational taxonomic units (OTUs) with uclust (Edgar, 2010), using a 97% similarity threshold. From each OTU, the most abundant sequence was selected as representative, and the taxonomy was assigned with the uclust-based consensus taxonomy assigner against the Greengenes database (version 13.8). The representative sequence set was aligned with PyNAST (Caporaso et al., 2010a), and potential chimeric sequences were discarded based on a check with ChimeraSlayer. OTU tables were constructed, and OTUs not assigned to the class of *Gammaproteobacteria* and singletons were removed from the dataset.

### Statistical Analysis

The datasets were normalized by rarefying to the lowest number of reads (3,351 high-quality reads) and MetagenomeSeq's cumulative sum scaling (Paulson et al., 2013) was used for alpha and beta diversity analysis, respectively. Phyloseq, MicrobiomeAnalyst, and vegan R packages implemented in RStudio v1.3.1093 were used to analyze bacterial community diversity and composition (Oksanen et al., 2007; Allaire, 2012; Core Team, 2013; McMurdie and Holmes, 2013; Dhariwal et al., 2017; Chong et al., 2020). Significant differences in alpha diversity based on Shannon index (H') were determined using ANOVA and Tukey-HSD *post hoc* test. The normalized weighted Unifrac dissimilarity matrix (Lozupone et al., 2011) was subjected to permutational ANOVA (PERMANOVA) to test for significant effects of factors on microbial community structure. The Adonis test with 999 permutations was used for pairwise comparisons. Biomarkers at the bacterial genus level between the tested factors were identified using a linear discriminant analysis effect size (LefSe) (Segata et al., 2011). The threshold of linear discriminant analysis was set as 1 with cutoff *p* values of 0.1.

## Results

After sequencing, a total of 6,550,680 quality reads and 72,861 OTUs were retrieved. Filtering singletons, chimeric and non-gammaproteobacterial reads left 5,011,782 high-quality reads that were retained and clustered into 17,824 gammaproteobacterial OTUs. Rarefaction curves indicate that the sequencing depth was sufficient to capture gammaproteobacterial diversity (Supplementary Figure S1).

### Microbial Alpha Diversity Is Shaped by Location, Microenvironment, and Treatment

**Figure 2:**
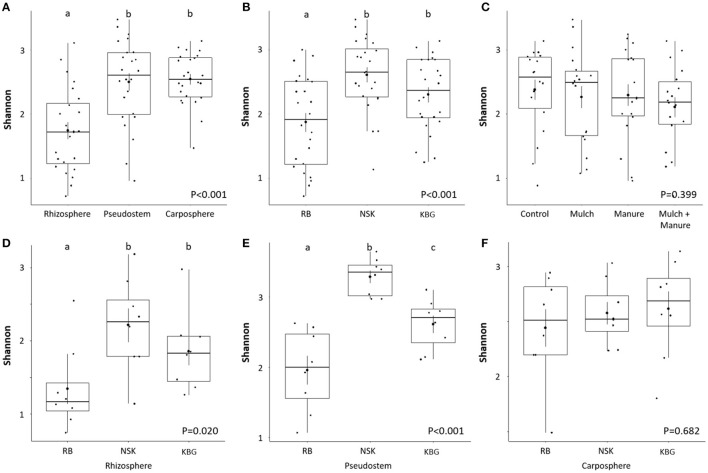
Shannon indices of gammaproteobacterial diversity in different microhabitats **(A)**, smallholder farms **(B)**, organic soil amendment treatments **(C)**, and for different microenvironments in different smallholder farms **(D–F)** at a genetic similarity level of 97%. Significant differences (*p* ≤ 0.05, ANOVA with Tukey-HSD *post hoc* test) within tested factors are indicated by different letters above the boxplot. Smallholder farms are abbreviated with RB, NSK, and KBG.

Analysis of variance indicated that microhabitat and sampling location influenced gammaproteobacterial diversity (*p* < 0.001), whereas different soil amendments showed unexpectedly no significant effect overall (*p* = 0.399). Microbial alpha diversity calculated based on the Shannon index showed a generally lower gammaproteobacterial diversity in rhizosphere samples compared to endo- and carposphere (*p* < 0.001; Figure 2A). Within smallholder farms, we identified a highly site-specific microbiome fingerprint: the lowest alpha diversity was observed in RB sampling sites (*p* < 0.001; Figure 2B). Systematical analysis was performed to determine significant differences among treatments. While there was no significant treatment effect on the alpha diversity overall (*p* = 0.399; Figure 2C), some significant treatment-based changes were observed when farms were analyzed separately. Soil amendment changed the gammaproteobacterial diversity in the endosphere of the farm NSK (*P* = 0.035, Supplementary Figure S2). The combination of mulch and manure increases the gammaproteobacterial diversity in comparison to the control (Supplementary Figure S2). Soil amendment also changed the gammaproteobacterial diversity in the rhizosphere and endosphere of KBG farm. However, these findings were only significant at the 90% confidence level (rhizosphere—*p* = 0.076 and carposphere—*p* = 0.077). Mulch treatment increased gammaproteobacterial diversity in the rhizosphere in comparison to control. Mulch, manure, and their combination showed a higher diversity in the carposphere of the KBG farm compared to the control (Supplementary Figure S2). Looking closer into differences within smallholder farms, the highest alpha diversities were found in the rhizo- and endosphere of NSK, while RB showed generally lower diversities (*p* < 0.001; Figures 2D–F).

### The Microbial Composition of Banana Plants Is Microhabitat Specific

**Figure 3:**
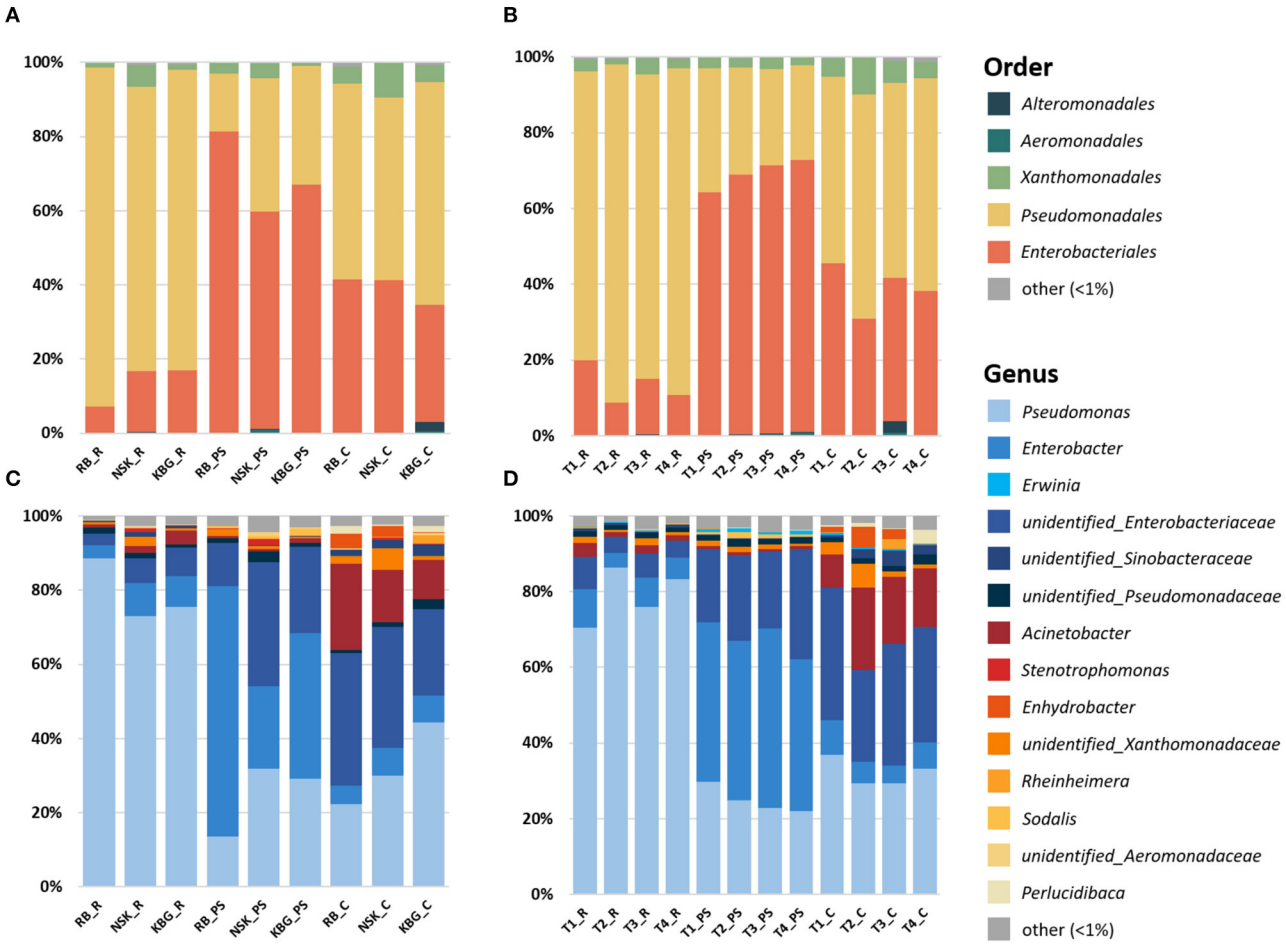
Taxonomic composition of *Gammaproteobacteria* communities inhabiting rhizosphere, pseudostem endosphere, and carposphere of banana plants grown at three smallholder farms in Uganda under varying organic management regimes. Sequences were classified at order **(A,B)** and genus **(C,D)** levels. Groups were encoded using abbreviations indicating: (1) farm (RB, NSK, KBG), (2) microenvironment (R, rhizosphere, PS, pseudostem endosphere, C, carposphere), and (3) organic matter input (T1 = mulch, T2 = mulch + manure, T3 = manure, T4 = non-treated).

Gammaproteobacterial community composition was analyzed on different phylogenetic levels. Already at the order level, differences between the three microhabitats (rhizosphere, endosphere, and carposphere) were visible (Figure 3A). The rhizosphere and carposphere were dominated by a major fraction of *Pseudomonadales* [76–92% (rhizosphere); 49–60% (carposphere), and 16–36% (pseudostem endosphere)]. In contrast, the pseudostem endosphere was dominated by a high fraction of *Enterobacteriales* (59–81%), which was also present in the rhizosphere (7–17%) and carposphere (32–41%). Moreover, minor fractions of *Xanthomonadales* were found in all three microenvironments (1–9%). At the genus level, *Pseudomonas* was dominating the rhizosphere (73–89%) with minor fractions of *Enterobacter* (3–9%) and an unidentified *Enterobacteriaceae* (2–8%). In the pseudostem endosphere, *Enterobacter* (22–67%), *Pseudomonas* (14–32%), and unidentified *Enterobacteriaceae* (12–34%) were present. In contrast, the carposphere showed, additionally to *Pseudomonas* (22–44%), *Enterobacter* (5–8%), and unidentified *Enterobacteriaceae* (23–36%), a high fraction of *Acinetobacter* (10–23%) (Figure 3C). If samples were grouped by organic matter treatment (non-treated, mulch, manure, mulch + manure), similar patterns between the three microenvironments were visible, however, major effects induced by treatments were not observed (Figures 3B,D). Mulch and manure treatment increased the abundance of *Enterobacteriales* from 11% (control) to 20% (mulch) and 15% (manure) in the rhizosphere. Moreover, mulch also increased the *Enterobacteriales* fraction in the carposphere [from 38% (control) to 45% (mulch)]. The abundance of *Pseudomonadales*, on the other hand, was decreased by manure and mulch treatment in parallel to the increase of *Enterobacteriales*. Contrastingly, in the pseudostem endosphere, all treatments decreased the abundance of *Enterobacteriales* and increased the abundance of *Pseudomonadales*. To investigate detailed changes induced by treatments on genus level LEfSe analysis was performed below.

### Microbial Community Structure and Taxonomic Changes Induced by Treatments

**Figure 4:**
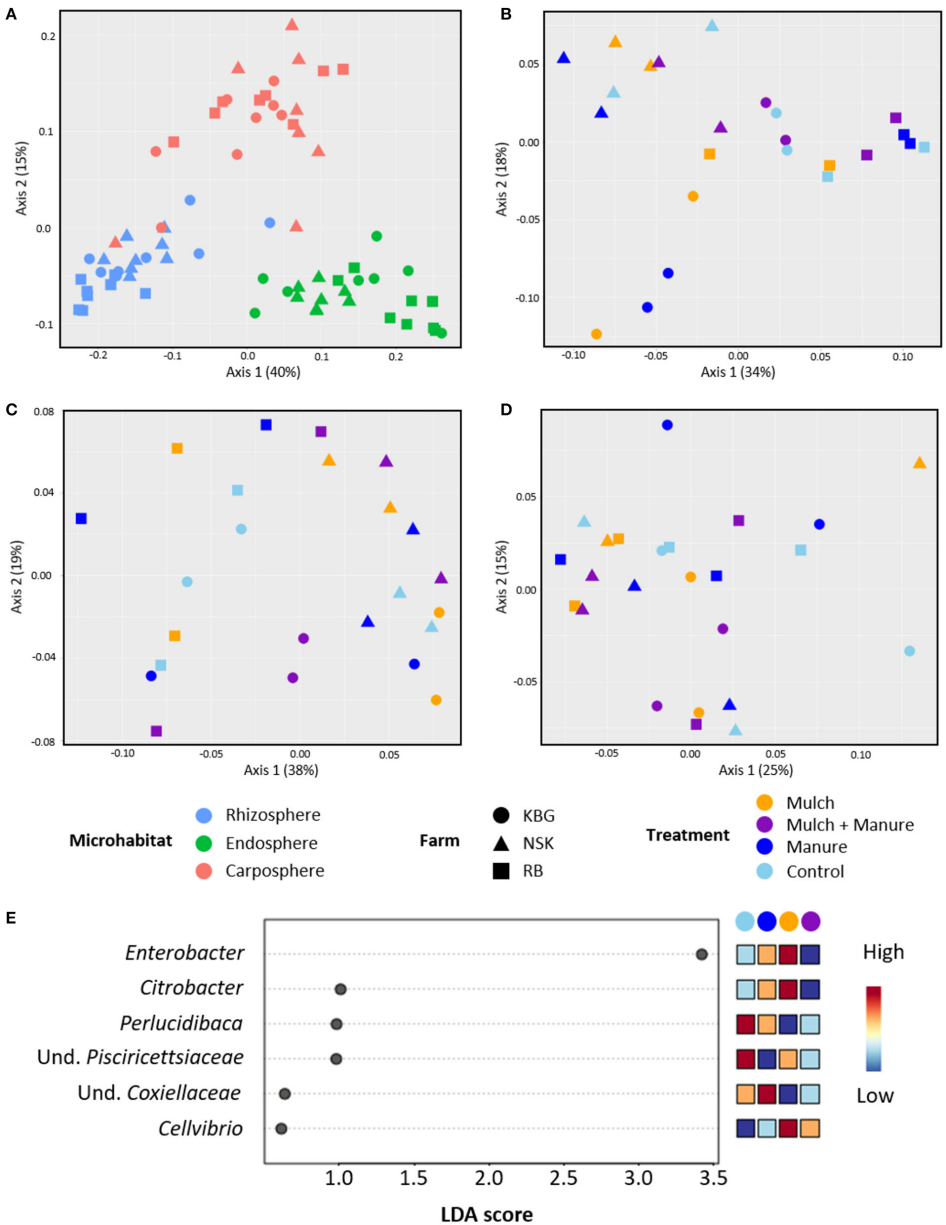
Principal coordinate analysis (PCoA) plots of the gammaproteobacterial communities inhabiting all microenvironments **(A)**, rhizosphere **(B)**, pseudostem endosphere **(C)**, and carposphere **(D)** of banana plants grown at three different farms (RB, NSK, and KBG) in Uganda under varying organic management regimes. Linear discriminant analysis effect size (LEfSe) analysis identifying considerably different taxa between treatments in the rhizosphere across all locations **(E)**. PCoA plots are based on weighted UniFrac distances. The plots indicate the grouping of samples by the farm (different shapes) and individual treatments (different colored symbols).

Beta diversity analysis indicated that microhabitat was the main factor that influenced the gammaproteobacterial community structure (*p* = 0.001; Table 1). This factor explained 43.9% (as shown by PERMANOVA) of total gammaproteobacterial community variation as indicated by separated clustering according to the microhabitat (Figure 4A; Table 1). Sampling sites also influenced the gammaproteobacterial community structure (*p* = 0.003) but only explained a small variation of the community (3.7%). To test if the variation of gammaproteobacterial communities in each microhabitat is influenced by soil amendment treatment and/or farm, deepening analysis by separating datasets from each microhabitat was performed. Principal coordinate analysis (PCoA) plots indicated that the gammaproteobacterial communities of banana plants showed significant grouping in rhizo- and endosphere by the different farms (Figures 4B,C; Table 1). However, there was no grouping for the carposphere (Figure 4D; Table 1).

**Table 1:** Permutational ANOVA (PERMANOVA) performed on the weighted Unifrac dissimilarity matrix showing influencing factors for the whole dataset and each microenvironment.

**Factor**	***P* value**	**R^**2**^ value**
**All datasets**		
Microenvironment	0.001	0.439
Farm	0.003	0.037
Treatment	0.538	0.018
		
**Rhizosphere datasets**		
Farm	0.004	0.282
Treatment	0.050	0.182
		
**Endosphere datasets**		
Farm	0.001	0.304
Treatment	0.712	0.077
		
**Carposphere datasets**		
Farm	0.651	0.076
Treatment	0.817	0.105

Soil amendment treatment only influenced the gammaproteobacterial community structure in the rhizosphere (*p* = 0.050, Supplementary Table S1). When pairwise comparisons using Adonis between control and each treatment were performed, a significant shift in gammaproteobacterial community structure was observed after amendments of mulch (*p* = 0.034) and manure (*p* = 0.034). Interestingly, a combinatory treatment (mulch and manure) did not affect the gammaproteobacterial community structure (*p* = 0.612). Subsequently, LEfSe analysis was conducted to identify taxa that were significantly affected by soil amendment treatments. Using a stringent *p* value, there was no bacterial genera significantly affected by treatment. However, when the stringency was reduced (from *p* > 0.01 to *p* < 0.1), LEfSe analysis indicated that four bacterial genera and two taxa that could not be classified into genus levels were affected by the treatments. These taxa belonged to *Enterobacter, Citrobacter, Perlucidibaca*, unclassified *Pisciricettsiaceae*, unidentified *Coxiellaceae*, and *Cellvibrio* (Figure 4E). The first two taxa were consistently enriched in the mulch as well as manure-treated samples. *Cellvibrio* was enriched in manure and in the combinatory manure and mulch treatment. Moreover, the abundances of *Perlucidibaca* and an unclassified *Pisciricettsiaceae* were higher in the control.

## Discussion

In this study, we investigated the effect of organic management regimes on the gammaproteobacterial community in the rhizo-, endo-, and carposphere of banana in three smallholder farms in Uganda. Previous results showed that nutrient input by organic matter, often produced from local sources, increases nutrient availability and has a tremendous effect on the achieved bunch sizes and yields (Mpiira et al., 2013; Staver et al., 2015). Nevertheless, our results show that treatments overall did not have a significant effect on gammaproteobacterial alpha diversity. This was in contrast to our expectations but indicates a generally stable gammaproteobacterial community. When farms were separated, significant differences induced by organic soil amendments were found. For instance, manure increased gammaproteobacterial diversity in the rhizosphere and carposphere in the farm KBG. Despite the fact, that the effect was specific for each of the three selected farms, still general microbiota patterns were identified.

Banana habitats (rhizosphere, pseudostem, and fruit) and their specific biotic and abiotic parameters were identified as the main drivers of the microbiota. Overall, gammaproteobacterial diversity was lower in the rhizosphere compared to endophytic aboveground parts (pseudostem and banana fruit). This is in contrast to most published studies about the plant microbiota, but confirms our previous study in banana, when we reported this unexpected finding for the first time (Rossmann et al., 2012). In addition, the diversity values observed for aboveground plant parts were comparable to our previous findings in other banana plantations in Central America (Köberl et al., 2015, 2017). Beta diversity grouping was mainly due to microhabitat, while effects of sampling farm and treatment were only visible when samples were separated by habitat first. Similarly to alpha diversity measures, the three farms RB, NSK, and KBG significantly differed in microbial composition in the rhizo- and endosphere, while no differences were found for the carposphere.

Soil amendments were found to influence the gammaproteobacterial community structure only in the rhizosphere. Further taxonomic composition analysis was in line with our previous observations. The microenvironment effect was more visible than the treatment effect. Confirming our expectations, an increase in *Enterobacteriales* abundance due to manure amendment was observed in the rhizosphere. Moreover, the mulch amendment did have this effect on the rhizo- and carposphere. Interestingly, combinatory treatments did not have such an effect. On the contrary, treatments had the opposite effect in the pseudostem endosphere, which was generally dominated by *Enterobacteriales*. Here, all treatments increased the abundance of *Pseudomonadales* and decreased *Enterobacteriales* fractions. Both *Pseudomonadales* and *Enterobacteriales* species were previously discovered to play a key role in plant-associated communities (Berg et al., 2014; Wassermann et al., 2017; Cernava et al., 2019) and are especially high in the pseudostem of banana (Rossmann et al., 2012; Nimusiima et al., 2015).

We highlight specifically that manure and mulch treatments in the soil affected both above- and belowground communities. The strong connectivity of soil microbial communities with the aboveground and especially fruit community was just recently discovered (Bergna et al., 2018; Kusstatscher et al., 2020). Microbial diversity in the soil, as well as management regime, has significant effects on carposphere communities (Abdelfattah et al., 2016; Wassermann et al., 2019). Moreover, *Gammaproteobacteria*, which can be usually found in high abundances within plant-associated communities, were previously discovered to be affected above- and belowground by agroforestry treatments (Fürnkranz et al., 2012; Köberl et al., 2015). In contrast to previous studies, we here analyzed also the effect of different organic soil inputs. While no major community shifts were induced by the applied treatments, LEfSe analysis elucidated considerable taxonomic shifts at the genus level. Interestingly, both *Enterobacter* and *Citrobacter* were significantly increased in the rhizosphere by manure and mulch treatments. Moreover, *Cellvibrio* was significantly increased by both manure and combinatory treatment (manure and mulch). All those taxa were previously discussed as members of the core microbiome of leafy greens, citrus fruits, grapevine, and banana (Zarraonaindia et al., 2015; Köberl et al., 2017; Xu et al., 2018; Cernava et al., 2019). *Enterobacter* was moreover associated with diseases, such as *F*. wilt, in banana but is at the same time a well-known antagonist and important keystone taxa against fungal diseases (Köberl et al., 2017; Liu et al., 2019).

To our knowledge, we were the first to specifically investigate the banana fruit microbiome. Interestingly, banana fruit harbored a richer and more balanced gammaproteobacterial community than the rhizo- and endosphere. Compositional analysis showed a higher relative abundance of *Pseudomonas* (22–44%), unidentified *Enterobacteriaceae* (23–36%), *Acinetobacter* (10–23%), and *Enterobacter* (5–8%). Studying the edible microbiome, e.g., microbiome of fruits, became recently a trend and is important to assess risks for human consumption as well as storability (Berg et al., 2015; Droby and Wisniewski, 2018; Kusstatscher et al., 2020). The group of *Enterobacteriaceae*, even though comprising a number of human pathogens, were previously shown to be part of the native fruit microbiome in mango and strawberry and harbor specific functions (Diskin et al., 2017; Zhang et al., 2020). Therefore, a higher abundance in banana fruit is not surprising.

Plant and soil management practices to change soil characteristics have been discussed as a potential strategy to prevent panama disease in banana (Mena et al., 2015; Omondi et al., 2020). Organic matter sourced from local shrubs and trees harbors a rich source of nitrogen, phosphorous and potassium, needed for banana production (Kongkijthavorn, 2017). While bunch size and yield could be improved (Staver et al., 2015), this study shows the high resilience of gammaproteobacterial communities in African smallholder cropping systems. Highly diverse soils were previously discussed to be more resistant to changes and can harbor suppressive characteristics (Raaijmakers and Mazzola, 2016). In this study even though organic amendments were applied over 2 years compared to a control without amendments, no major shifts but only changes in a low number of taxonomic groups were observed. Our previous studies showed that major shifts are associated with plant diseases, however healthy banana plants were associated with similar communities independently from their cropping system or planting location (Köberl et al., 2015, 2017). Similarly, Wemheuer et al. (2020) showed the stability of bacterial communities in cocoa agroforests, while fungal communities changed (Wemheuer et al., 2020). Taken together, we here, confirmed former observations and moreover, increased our knowledge of the complex banana agroecosystem. Microbiome management in the soil will be of significant importance to maintain healthy agricultural soils, not only in banana farming. Even though gammaproteobacterial communities were shown to be stable under multiple conditions, future investigations including the whole bacterial as well as multi-kingdom communities should give a better look into soil amendments impact on the banana-associated microbial community. Organic amendments applied over a longer period also deserve attention.

## Data Availability Statement

The datasets presented in this study can be found in online repositories. The names of the repository/repositories and accession number(s) can be found below: https://www.ebi.ac.uk/ena, PRJEB48209.

## Author Contributions

MK, CS, and GB designed the study. SM and FK conducted the fields experiment and collected the samples. MK, PK, and WW analyzed the data and wrote the manuscript. CS and GB critically reviewed the manuscript. All authors approved the final paper.

## Funding

This study was supported by the Federal Ministry for Europe, Integration and Foreign Affairs (BMEIA) of the Republic of Austria through the Austrian Development Agency (ADA), and the CGIAR Research Program on Roots, Tubers, and Bananas (CRP RTB). MK acknowledges support from the Austrian Science Fund FWF (T 847).

## Conflict of Interest

The authors declare that the research was conducted in the absence of any commercial or financial relationships that could be construed as a potential conflict of interest.

## Publisher's Note

All claims expressed in this article are solely those of the authors and do not necessarily represent those of their affiliated organizations, or those of the publisher, the editors and the reviewers. Any product that may be evaluated in this article, or claim that may be made by its manufacturer, is not guaranteed or endorsed by the publisher.
