# Supplementary material for: Increased Yield and High Resilience of Microbiota Representatives With Organic Soil Amendments in Smallholder Farms of Uganda
Source: Front Plant Sci. 2022 Feb 2;12:815377. doi: 10.3389/fpls.2021.815377 (PMC8847376; doi:10.3389/fpls.2021.815377)
Supplement: Supplementary file 1 [file Table_1.DOCX]

Supplementary Material

# Supplementary Figures and Tables


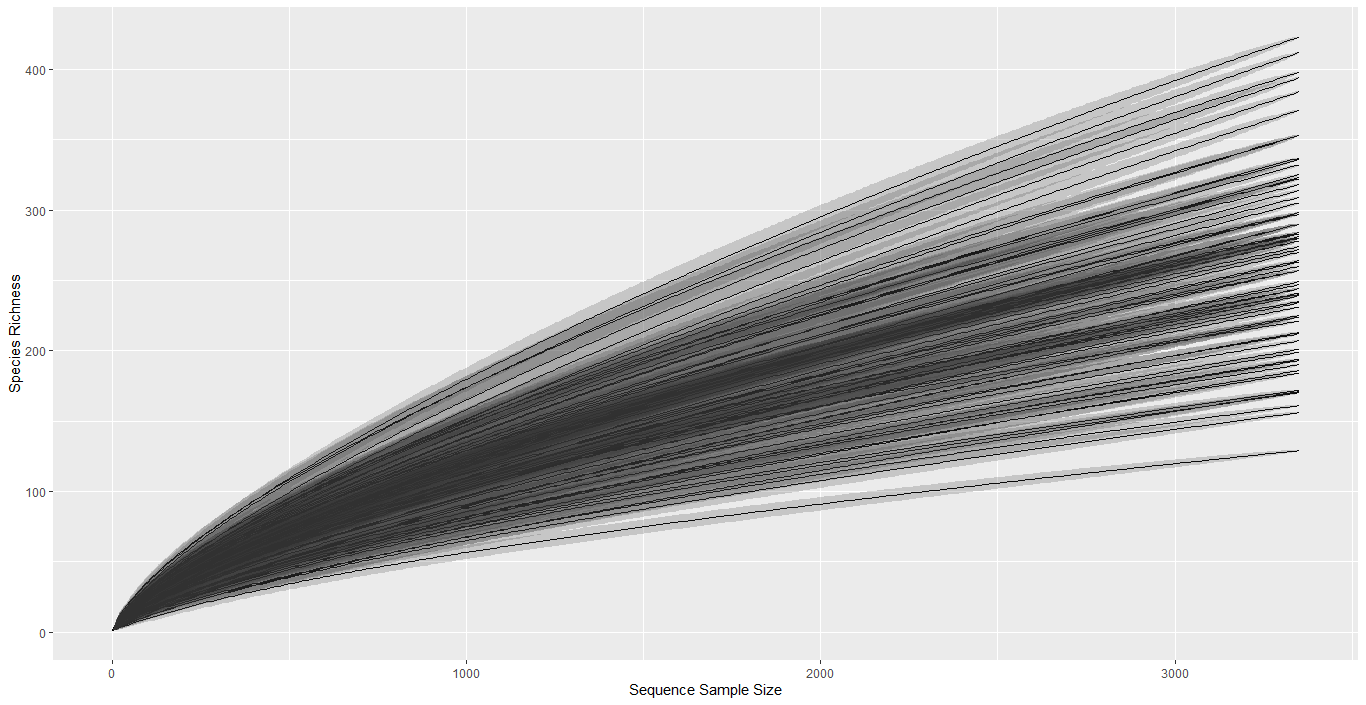


Figure S1. Rarefaction curves indicating that the sequencing depth is sufficient to capture gammaproteobacterial diversity.


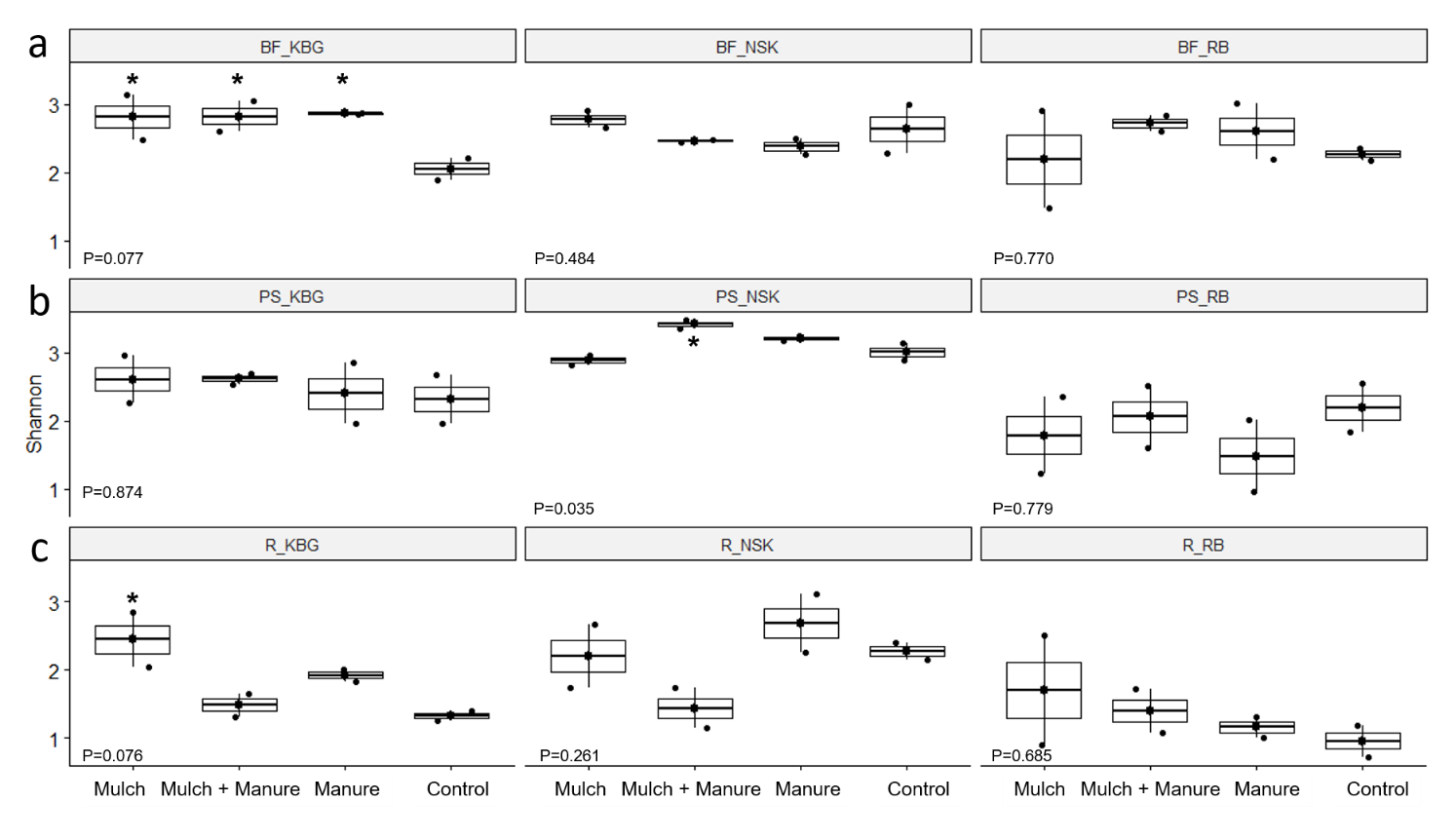


Figure S2. Alpha diversity measures given in Shannon diversity for each microhabitat (carposphere (A), pseudostem endosphere (B) and rhizosphere (C)) and farm (KBG, NSK, RB). Significant differences between treatments and control were tested using ANOVA and are indicated with asterisks.


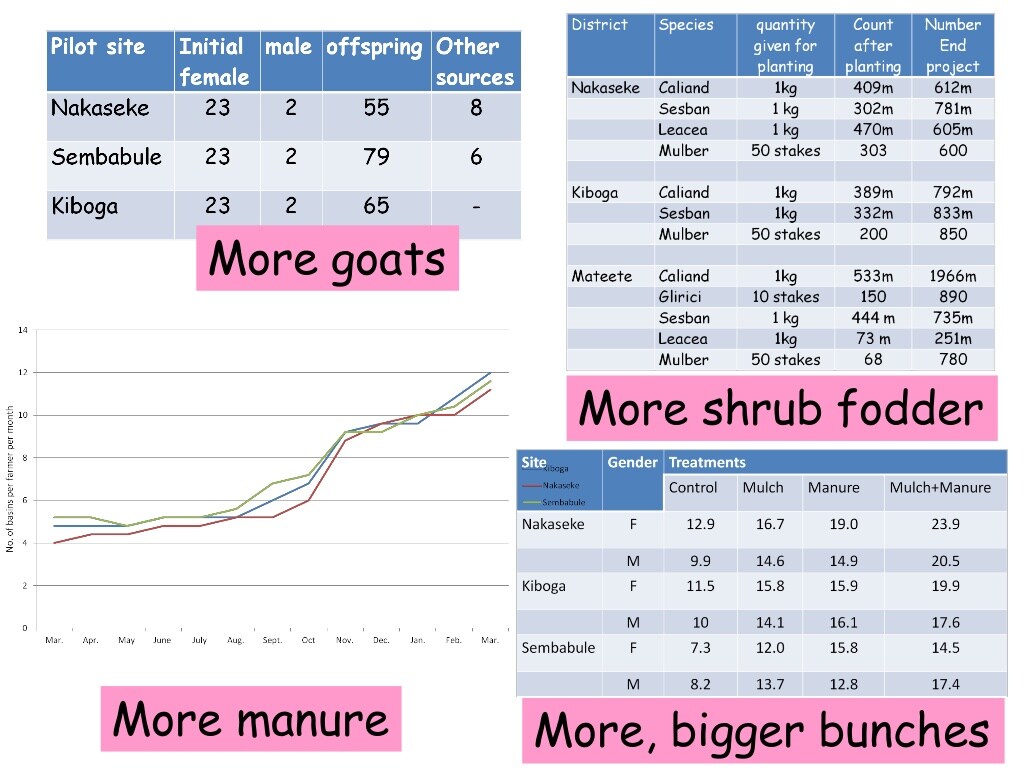


Figure S3. Key figure from Staver et al. (2015) showing the increased banana yield following organic soil amendments.

Table S1. PERMANOVA analysis performed on the weighted Unifrac dissimilarity matrix showing influence of treatments on different farms in the rhizosphere.

| Farm | PERMANOVA | |
| --- | --- | --- |
|  | R^2^ value | P value |
| NSK | 0.552 | 0.304 |
| RB | 0.330 | 0.799 |
| KBG | 0.683 | 0.021* |
